# 2-*tert*-Butyl-6-(cyclo­hexyl­imino­meth­yl)-4-meth­oxy­phenol

**DOI:** 10.1107/S1600536811023385

**Published:** 2011-06-22

**Authors:** Roghayieh Jamjah, Mehdi Nekoomanesh, Tayebeh Pourjafar, Gholam Hossein Zohuri, Faramarz Afshartaromi, Behrouz Notash

**Affiliations:** a Department of Catalyst Polymerization Engineering Faculty, Iran Polymer and Petrochemical Institute (ippi), PO Box 14965/115, 14185/485, Tehran, Iran; b Chemistry Group Amirkabir University, Tehran, Iran; c Department of Chemistry, Ferdowsi University of Mashhad, Mashhad,, Iran; d Department of Chemistry, Shahid Beheshti University, G. C., Evin, Tehran 1983963113, Iran

## Abstract

The asymmetric unit of the title Schiff base compound, C_18_H_27_NO_2_, contains two independent mol­ecules in which the C=N bond lengths are 1.278 (2) and 1.280 (2) Å and the cyclo­hexane rings adopt chair conformations. Intra­molecular O—H⋯N hydrogen bonding between hy­droxy and imine groups and weak C—H⋯O hydrogen bonds help to stabilize the mol­ecular structure.

## Related literature

For general background to the synthesis and catalytic activity of the FI family of early transition metal olefin polymerization catalysts, see: Matsui & Fujita (2001 ▶); Matsui *et al.* (1999 ▶, 2001 ▶); Makio *et al.* (2002 ▶); Suzuki *et al.* (2006 ▶); Saito *et al.* (2002 ▶); Parssinen *et al.* (2005 ▶). For background to the synthesis of Schiff base compounds, see: Hofsløkkn & Skattebøl (1999 ▶); Wang *et al.* (1994 ▶); Gregson *et al.* (2006 ▶); Bigi *et al.* (2000 ▶). For the synthesis of phen­oxy-imine ligands and their complexes, see: Matsukawa *et al.* (2001 ▶); Tohi *et al.* (2004 ▶); Makio *et al.* (2002 ▶). For related structures, see: Hiller *et al.* (1993 ▶); Darensbourg *et al.* (2005 ▶).
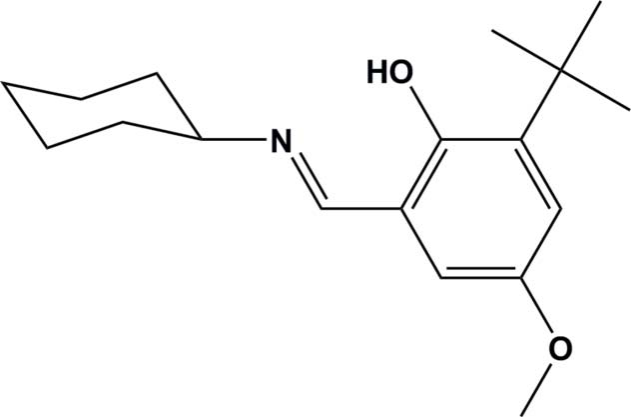


## Experimental

### 

#### Crystal data

C_18_H_27_NO_2_
*M* *_r_* = 289.41
Triclinic, 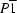

*a* = 10.388 (2) Å
*b* = 13.325 (3) Å
*c* = 13.766 (3) Å
α = 111.37 (3)°
β = 108.31 (3)°
γ = 92.46 (3)°
*V* = 1657.8 (8) Å^3^
*Z* = 4
Mo *K*α radiation
μ = 0.07 mm^−1^
*T* = 120 K
0.45 × 0.45 × 0.30 mm

#### Data collection

Stoe IPDS II diffractometer
18474 measured reflections
8861 independent reflections
6731 reflections with *I* > 2σ(*I*)
*R*_int_ = 0.095

#### Refinement

*R*[*F* ^2^ > 2σ(*F* ^2^)] = 0.059
*wR*(*F* ^2^) = 0.209
*S* = 1.09
8861 reflections
395 parameters
H atoms treated by a mixture of independent and constrained refinement
Δρ_max_ = 0.59 e Å^−3^
Δρ_min_ = −0.51 e Å^−3^

### 

Data collection: *X-AREA* (Stoe & Cie, 2005 ▶); cell refinement: *X-AREA*; data reduction: *X-AREA*; program(s) used to solve structure: *SHELXS97* (Sheldrick, 2008 ▶); program(s) used to refine structure: *SHELXL97* (Sheldrick, 2008 ▶); molecular graphics: *ORTEP-3 for Windows* (Farrugia, 1997 ▶); software used to prepare material for publication: *WinGX* (Farrugia, 1999 ▶).

## Supplementary Material

Crystal structure: contains datablock(s) I, global. DOI: 10.1107/S1600536811023385/xu5239sup1.cif

Structure factors: contains datablock(s) I. DOI: 10.1107/S1600536811023385/xu5239Isup2.hkl

Supplementary material file. DOI: 10.1107/S1600536811023385/xu5239Isup3.cml

Additional supplementary materials:  crystallographic information; 3D view; checkCIF report

## Figures and Tables

**Table 1:** Hydrogen-bond geometry (Å, °)

*D*—H⋯*A*	*D*—H	H⋯*A*	*D*⋯*A*	*D*—H⋯*A*
O1—H1⋯N1	0.88 (3)	1.77 (3)	2.5918 (19)	156 (3)
O3—H2⋯N2	0.90 (3)	1.73 (3)	2.5901 (19)	159 (3)
C5—H5*B*⋯O1	0.96	2.34	2.994 (2)	125
C6—H6*B*⋯O1	0.96	2.36	3.004 (2)	124
C23—H23*B*⋯O3	0.96	2.41	3.051 (2)	124
C24—H24*B*⋯O3	0.96	2.36	3.000 (2)	124

**Fig. 1.**
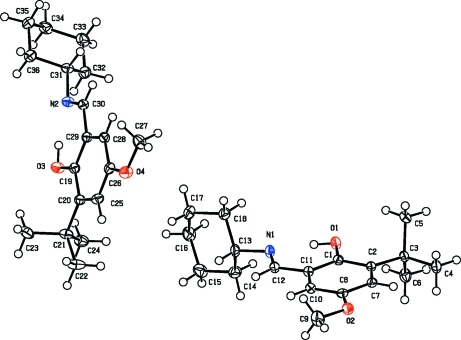
The molecular structure of the title compound with displacement ellipsoids drawn at 50% probability level.

**Fig. 2.**
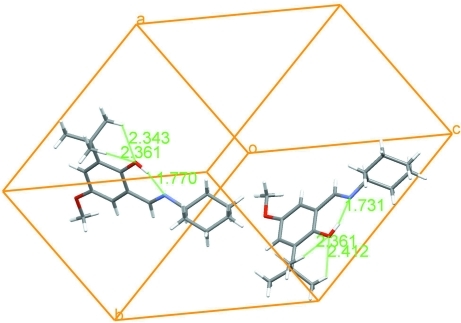
The intramolecular C—H···O and O—H···N hydrogen bonds are shown as green dashed lines.

## supplementary crystallographic information

### Comment

In the late 1990s' Fujita group discovered and developed a new family of early transition metal catalysts [FI catalysts] (Matsui & Fujita, 2001; Matsui *et al.*, 2001; Makio *et al.*, 2002). These new catalysts with two phenoxy-imine chelate ligands were discovered on the basis of, ligand oriented catalyst design concept, and show high activity for olefin polymerization (Matsui *et al.*, 1999; Suzuki *et al.*, 2006).

FI catalysts can produce a wide variety of new polymers whose are comparable to those produced by group 4 metallocen catalysts which are unobtainable with conventional Ziegler-Natta catalysts. FI catalysts are generally comprised of transition metals (Zr, Ti, *etc*) (Suzuki *et al.*, 2006) and ligand(*s*) with general formula of *L*_2_M*X*_2_ (*M*= Transition metal, *L* = ancillary ligand(*s*), and *X*= monodentate anionic ligand such as halide or amide (Matsui & Fujita, 2001; Saito *et al.*, 2002; Parssinen *et al.*, 2005). The basic phenoxy-imine ligand systems can be divided into two bases reactant: primary amines and salicylaldehyde derivatives.

Usually amines and some salicylaldehyde derivatives commercially are available, but some of them such as 2-hydroxy-3-*tert*-butyl-5-methoxy benzaldehyde and the ones with desired substituents are not commercially available and can be synthesized by straight forward synthetic methods. Formylation at the 2-position of phenols can be performed using paraformaldehyde with many established methods in high yields (typically 70–80%). Electron donating substitueants such as methoxy group at the *para* position of phenoxy oxygen in benzene ring enhance the rate of formulation reaction. Salicylaldehydes and primary amines are condensed into Schiff bases under standard condensation condition which can obtain with high selectivity and yields (Hofsløkkn & Skattebøl, 1999; Wang *et al.*, 1994; Gregson *et al.*, 2006; Bigi *et al.*, 2000). Generally, the overall synthesis requires fewer steps and gives higher yield than those for metallocences. Rational design of the phenoxy-imine ligand and its effect on activity, thermal stability and molecular weight capabilities and molecular weight distributions that could be achieved by varying combination of *R*_1_, *R*_2_ and *R*_3_ groups on the final ligand (Matsukawa *et al.*, 2001; Tohi *et al.*, 2004). Once again, designing the ligand frame work by addition of an electron-donating group in the *R*_3_ position, can be impart a large electronic influence on the Zirconium and strengthening the metal-ligand interactions (Makio *et al.*, 2002).

Herein, we report synthesis and crystal structure of new schiff base compound ((*E*)-2-*tert*-butyl-6-((cyclohexylimino)methyl)-4-methoxyphenol). The asymmetric unit of the title compound is shown in Fig. 1 and contain two molecules of schiff base compound. The bond lengths and angles are comparable to those observed for schiff base ligands (Hiller *et al.*, 1993; Darensbourg *et al.*, 2005). In the crystal structure of title compound, there is intramolecular bifurcated C—H···O hydrogen bondings between two methyl from t-buthyl group and hydroxy group and also intramolecular O—H···N between hydroxy and nitrogen of imine part (Table 1 & Fig. 2).

### Experimental

Ligand synthesis was carried out under an atmosphere of nitrogen using oven-dried glassware. To a 100 ml flask thoroughly purged with nitrogen, 30 ml of ethanol, 1.90 g (12.0 mmol) of dried and fresh distilled cyclohexylamine and 2.08 g (10.0 mmol) of 5-methoxy 3 - t-butylsalicylaldehyde were introduced. After addition of 5 g of activated molecular sieve 3 Å, the mixture was stirred at room temperature for 12 h and then filtered. The molecular sieve 3 Å was washed with ethyl acetate (20 ml). The combined organic filtrates were concentrated in vacuum to afford a crude imine compound. Reaction solution was concentrated under reduced pressure and yellow salicylaldimine obtained. Then the product recrystallized with petroleum ether (m.p. 90°C).

### Refinement

Hydroxy H atoms were found in a difference Fourier map and refined isotropically without restraint. Other H atoms were positioned geometrically and refined as riding atoms with C—H = 0.93 to 0.97 Å, *U*_iso_(H) = 1.5*U*_eq_(C) for methyl H atoms and 1.2*U*_eq_(C) for the others.

### Crystal data

**Table:**

C_18_H_27_NO_2_	*Z* = 4
*M**_r_* = 289.41	*F*(000) = 632
Triclinic, *P*1	*D*_x_ = 1.160 Mg m^−^^3^
Hall symbol: -P 1	Mo *K*α radiation, λ = 0.71073 Å
*a* = 10.388 (2) Å	Cell parameters from 8861 reflections
*b* = 13.325 (3) Å	θ = 2.2–29.2°
*c* = 13.766 (3) Å	µ = 0.07 mm^−^^1^
α = 111.37 (3)°	*T* = 120 K
β = 108.31 (3)°	Block, yellow
γ = 92.46 (3)°	0.45 × 0.45 × 0.30 mm
*V* = 1657.8 (8) Å^3^	

### Data collection

**Table:**

Stoe IPDS II diffractometer	6731 reflections with *I* > 2σ(*I*)
Radiation source: fine-focus sealed tube	*R*_int_ = 0.095
graphite	θ_max_ = 29.2°, θ_min_ = 2.2°
rotation method scans	*h* = −14→14
18474 measured reflections	*k* = −18→18
8861 independent reflections	*l* = −18→17

### Refinement

**Table:**

Refinement on *F*^2^	Primary atom site location: structure-invariant direct methods
Least-squares matrix: full	Secondary atom site location: difference Fourier map
*R*[*F*^2^ > 2σ(*F*^2^)] = 0.059	Hydrogen site location: inferred from neighbouring sites
*wR*(*F*^2^) = 0.209	H atoms treated by a mixture of independent and constrained refinement
*S* = 1.09	*w* = 1/[σ^2^(*F*_o_^2^) + (0.1285*P*)^2^ + 0.3083*P*] where *P* = (*F*_o_^2^ + 2*F*_c_^2^)/3
8861 reflections	(Δ/σ)_max_ < 0.001
395 parameters	Δρ_max_ = 0.59 e Å^−^^3^
0 restraints	Δρ_min_ = −0.51 e Å^−^^3^

### Special details

**Table:**

Geometry. All e.s.d.'s (except the e.s.d. in the dihedral angle between two l.s. planes) are estimated using the full covariance matrix. The cell e.s.d.'s are taken into account individually in the estimation of e.s.d.'s in distances, angles and torsion angles; correlations between e.s.d.'s in cell parameters are only used when they are defined by crystal symmetry. An approximate (isotropic) treatment of cell e.s.d.'s is used for estimating e.s.d.'s involving l.s. planes.
Refinement. Refinement of *F*^2^ against ALL reflections. The weighted *R*-factor *wR* and goodness of fit *S* are based on *F*^2^, conventional *R*-factors *R* are based on *F*, with *F* set to zero for negative *F*^2^. The threshold expression of *F*^2^ > σ(*F*^2^) is used only for calculating *R*-factors(gt) *etc*. and is not relevant to the choice of reflections for refinement. *R*-factors based on *F*^2^ are statistically about twice as large as those based on *F*, and *R*- factors based on ALL data will be even larger.

### Fractional atomic coordinates and isotropic or equivalent isotropic displacement parameters (Å^2^)

**Table:**

	*x*	*y*	*z*	*U*_iso_*/*U*_eq_	
C16	0.5193 (2)	0.86759 (17)	0.75550 (16)	0.0300 (4)	
H16A	0.6094	0.8661	0.8040	0.036*	
H16B	0.4585	0.8812	0.7980	0.036*	
C15	0.5309 (2)	0.95981 (16)	0.71639 (17)	0.0299 (4)	
H15A	0.5737	1.0281	0.7808	0.036*	
H15B	0.4393	0.9677	0.6764	0.036*	
C22	0.0841 (2)	0.92025 (15)	0.75356 (17)	0.0331 (4)	
H22A	0.0078	0.8755	0.6875	0.050*	
H22B	0.1657	0.9235	0.7354	0.050*	
H22C	0.0647	0.9928	0.7831	0.050*	
C23	−0.02620 (19)	0.86686 (15)	0.86736 (18)	0.0288 (4)	
H23A	−0.0458	0.9396	0.8948	0.043*	
H23B	−0.0145	0.8374	0.9230	0.043*	
H23C	−0.1013	0.8213	0.8008	0.043*	
C9	0.5171 (2)	0.77887 (18)	−0.01564 (17)	0.0305 (4)	
H9A	0.4676	0.7175	−0.0137	0.046*	
H9B	0.4935	0.7714	−0.0915	0.046*	
H9C	0.4929	0.8453	0.0267	0.046*	
C24	0.22712 (19)	0.94703 (14)	0.94596 (15)	0.0252 (4)	
H24A	0.3098	0.9478	0.9288	0.038*	
H24B	0.2397	0.9210	1.0042	0.038*	
H24C	0.2070	1.0198	0.9702	0.038*	
C34	0.3333 (2)	0.47319 (16)	1.27358 (16)	0.0276 (4)	
H34A	0.3090	0.5397	1.3176	0.033*	
H34B	0.3644	0.4326	1.3199	0.033*	
C17	0.4637 (2)	0.75723 (16)	0.65698 (17)	0.0282 (4)	
H17A	0.3696	0.7558	0.6131	0.034*	
H17B	0.4631	0.6997	0.6843	0.034*	
C21	0.10645 (18)	0.87064 (13)	0.84100 (14)	0.0211 (3)	
C35	0.2062 (2)	0.40417 (15)	1.17212 (16)	0.0258 (4)	
H35A	0.1321	0.3905	1.1969	0.031*	
H35B	0.2277	0.3341	1.1328	0.031*	
C33	0.44981 (19)	0.50278 (16)	1.23801 (16)	0.0267 (4)	
H33A	0.5266	0.5506	1.3035	0.032*	
H33B	0.4815	0.4367	1.2016	0.032*	
C14	0.6155 (2)	0.93769 (14)	0.64054 (16)	0.0252 (4)	
H14A	0.7104	0.9396	0.6834	0.030*	
H14B	0.6144	0.9950	0.6127	0.030*	
C18	0.55131 (19)	0.73550 (14)	0.58295 (15)	0.0229 (3)	
H18A	0.6434	0.7303	0.6249	0.028*	
H18B	0.5113	0.6662	0.5194	0.028*	
C5	1.10265 (18)	0.69749 (15)	0.36589 (16)	0.0242 (3)	
H5A	1.0633	0.6314	0.2998	0.036*	
H5B	1.0630	0.6974	0.4201	0.036*	
H5C	1.2005	0.7016	0.3960	0.036*	
C4	1.13992 (17)	0.79552 (14)	0.25165 (15)	0.0231 (3)	
H4A	1.1003	0.7297	0.1853	0.035*	
H4B	1.2372	0.7980	0.2832	0.035*	
H4C	1.1244	0.8580	0.2333	0.035*	
C32	0.40216 (18)	0.55994 (14)	1.15850 (15)	0.0231 (3)	
H32A	0.4763	0.5732	1.1336	0.028*	
H32B	0.3815	0.6303	1.1981	0.028*	
C6	1.13788 (18)	0.90406 (15)	0.44140 (15)	0.0245 (4)	
H6A	1.2357	0.9078	0.4705	0.037*	
H6B	1.0997	0.9055	0.4969	0.037*	
H6C	1.1188	0.9656	0.4221	0.037*	
C36	0.15883 (17)	0.46204 (14)	1.09267 (15)	0.0220 (3)	
H36A	0.0821	0.4143	1.0271	0.026*	
H36B	0.1269	0.5279	1.1293	0.026*	
C13	0.55966 (17)	0.82658 (14)	0.54196 (14)	0.0197 (3)	
H13	0.4673	0.8274	0.4944	0.024*	
C31	0.27464 (17)	0.49260 (13)	1.05690 (13)	0.0187 (3)	
H31	0.2986	0.4256	1.0114	0.022*	
C12	0.60610 (16)	0.80873 (13)	0.37989 (13)	0.0175 (3)	
H12	0.5148	0.8157	0.3509	0.021*	
C2	0.91611 (15)	0.79247 (12)	0.28903 (13)	0.0157 (3)	
C20	0.13799 (16)	0.75429 (12)	0.79790 (14)	0.0176 (3)	
C3	1.07256 (16)	0.79689 (13)	0.33652 (14)	0.0180 (3)	
C28	0.18954 (16)	0.53761 (13)	0.71137 (13)	0.0175 (3)	
H28	0.2065	0.4667	0.6831	0.021*	
C8	0.70950 (16)	0.78842 (12)	0.13850 (13)	0.0165 (3)	
C7	0.85104 (16)	0.78786 (12)	0.18216 (13)	0.0164 (3)	
H7	0.9035	0.7843	0.1376	0.020*	
C19	0.16492 (16)	0.69245 (12)	0.86441 (13)	0.0168 (3)	
C25	0.13920 (17)	0.70439 (13)	0.69027 (14)	0.0198 (3)	
H25	0.1234	0.7440	0.6456	0.024*	
C26	0.16305 (17)	0.59721 (13)	0.64589 (13)	0.0184 (3)	
C30	0.22574 (16)	0.52199 (13)	0.89036 (14)	0.0180 (3)	
H30	0.2456	0.4525	0.8597	0.022*	
C29	0.19070 (16)	0.58498 (12)	0.82116 (13)	0.0166 (3)	
C10	0.63036 (16)	0.79534 (12)	0.20365 (13)	0.0172 (3)	
H10	0.5371	0.7979	0.1764	0.021*	
C11	0.69178 (16)	0.79842 (12)	0.31176 (13)	0.0165 (3)	
C1	0.83340 (15)	0.79518 (12)	0.35442 (13)	0.0159 (3)	
O2	0.66130 (12)	0.78253 (10)	0.03121 (10)	0.0212 (3)	
O3	0.16646 (14)	0.73745 (10)	0.97098 (10)	0.0220 (3)	
O1	0.89028 (12)	0.79569 (10)	0.45748 (10)	0.0209 (3)	
O4	0.15796 (14)	0.56103 (10)	0.53771 (10)	0.0244 (3)	
N2	0.23021 (15)	0.55850 (11)	0.99129 (12)	0.0199 (3)	
N1	0.65112 (14)	0.80861 (12)	0.47775 (12)	0.0195 (3)	
C27	0.1861 (2)	0.45401 (15)	0.49089 (15)	0.0269 (4)	
H27A	0.2804	0.4526	0.5289	0.040*	
H27B	0.1701	0.4344	0.4132	0.040*	
H27C	0.1266	0.4028	0.4990	0.040*	
H2	0.189 (3)	0.684 (3)	0.995 (3)	0.055 (9)*	
H1	0.824 (3)	0.806 (2)	0.485 (2)	0.042 (7)*	

### Atomic displacement parameters (Å^2^)

**Table:**

	*U*^11^	*U*^22^	*U*^33^	*U*^12^	*U*^13^	*U*^23^
C16	0.0371 (10)	0.0400 (10)	0.0257 (9)	0.0166 (8)	0.0208 (8)	0.0178 (8)
C15	0.0407 (11)	0.0292 (9)	0.0264 (9)	0.0154 (8)	0.0195 (8)	0.0109 (7)
C22	0.0500 (12)	0.0195 (8)	0.0288 (9)	0.0098 (8)	0.0078 (8)	0.0134 (7)
C23	0.0243 (8)	0.0204 (8)	0.0370 (10)	0.0088 (6)	0.0076 (7)	0.0086 (7)
C9	0.0233 (9)	0.0467 (11)	0.0256 (9)	0.0104 (8)	0.0059 (7)	0.0208 (8)
C24	0.0262 (8)	0.0179 (7)	0.0251 (8)	0.0022 (6)	0.0044 (7)	0.0059 (6)
C34	0.0337 (9)	0.0342 (9)	0.0250 (9)	0.0167 (8)	0.0136 (7)	0.0188 (8)
C17	0.0282 (9)	0.0335 (9)	0.0332 (10)	0.0061 (7)	0.0178 (8)	0.0186 (8)
C21	0.0248 (8)	0.0148 (7)	0.0202 (8)	0.0044 (6)	0.0040 (6)	0.0063 (6)
C35	0.0285 (9)	0.0285 (8)	0.0302 (9)	0.0068 (7)	0.0149 (7)	0.0184 (7)
C33	0.0214 (8)	0.0331 (9)	0.0257 (9)	0.0088 (7)	0.0038 (7)	0.0152 (7)
C14	0.0312 (9)	0.0206 (7)	0.0274 (9)	0.0050 (6)	0.0153 (7)	0.0093 (7)
C18	0.0258 (8)	0.0201 (7)	0.0259 (8)	0.0044 (6)	0.0141 (7)	0.0082 (6)
C5	0.0206 (8)	0.0254 (8)	0.0324 (9)	0.0113 (6)	0.0111 (7)	0.0157 (7)
C4	0.0168 (7)	0.0261 (8)	0.0281 (9)	0.0048 (6)	0.0106 (6)	0.0101 (7)
C32	0.0199 (8)	0.0242 (8)	0.0255 (8)	0.0023 (6)	0.0056 (6)	0.0124 (7)
C6	0.0172 (7)	0.0251 (8)	0.0245 (8)	0.0007 (6)	0.0053 (6)	0.0047 (7)
C36	0.0190 (7)	0.0246 (8)	0.0221 (8)	0.0013 (6)	0.0043 (6)	0.0118 (6)
C13	0.0175 (7)	0.0255 (8)	0.0209 (8)	0.0067 (6)	0.0111 (6)	0.0104 (6)
C31	0.0232 (7)	0.0167 (7)	0.0177 (7)	0.0060 (6)	0.0069 (6)	0.0083 (6)
C12	0.0157 (7)	0.0184 (7)	0.0193 (7)	0.0037 (5)	0.0073 (6)	0.0073 (6)
C2	0.0143 (6)	0.0136 (6)	0.0196 (7)	0.0027 (5)	0.0068 (6)	0.0063 (5)
C20	0.0163 (7)	0.0160 (7)	0.0203 (7)	0.0028 (5)	0.0046 (6)	0.0086 (6)
C3	0.0137 (7)	0.0174 (7)	0.0233 (8)	0.0041 (5)	0.0075 (6)	0.0076 (6)
C28	0.0168 (7)	0.0165 (7)	0.0190 (7)	0.0032 (5)	0.0068 (6)	0.0063 (6)
C8	0.0195 (7)	0.0151 (6)	0.0167 (7)	0.0038 (5)	0.0063 (6)	0.0083 (5)
C7	0.0186 (7)	0.0136 (6)	0.0193 (7)	0.0036 (5)	0.0095 (6)	0.0067 (5)
C19	0.0160 (7)	0.0171 (7)	0.0166 (7)	0.0026 (5)	0.0053 (5)	0.0063 (6)
C25	0.0215 (7)	0.0186 (7)	0.0200 (8)	0.0021 (6)	0.0055 (6)	0.0103 (6)
C26	0.0196 (7)	0.0200 (7)	0.0172 (7)	0.0023 (6)	0.0079 (6)	0.0081 (6)
C30	0.0165 (7)	0.0164 (6)	0.0216 (7)	0.0028 (5)	0.0065 (6)	0.0084 (6)
C29	0.0145 (6)	0.0166 (7)	0.0192 (7)	0.0023 (5)	0.0050 (5)	0.0084 (6)
C10	0.0163 (7)	0.0171 (7)	0.0189 (7)	0.0042 (5)	0.0065 (6)	0.0076 (6)
C11	0.0158 (7)	0.0168 (7)	0.0179 (7)	0.0032 (5)	0.0071 (6)	0.0072 (6)
C1	0.0155 (7)	0.0156 (6)	0.0155 (7)	0.0030 (5)	0.0045 (5)	0.0060 (5)
O2	0.0212 (6)	0.0271 (6)	0.0199 (6)	0.0070 (5)	0.0079 (5)	0.0134 (5)
O3	0.0317 (7)	0.0204 (6)	0.0172 (6)	0.0101 (5)	0.0109 (5)	0.0085 (5)
O1	0.0168 (5)	0.0301 (6)	0.0193 (6)	0.0075 (5)	0.0079 (5)	0.0121 (5)
O4	0.0354 (7)	0.0228 (6)	0.0183 (6)	0.0062 (5)	0.0132 (5)	0.0087 (5)
N2	0.0233 (7)	0.0183 (6)	0.0202 (7)	0.0054 (5)	0.0073 (5)	0.0100 (5)
N1	0.0165 (6)	0.0246 (7)	0.0202 (7)	0.0049 (5)	0.0095 (5)	0.0095 (5)
C27	0.0298 (9)	0.0298 (9)	0.0209 (8)	0.0104 (7)	0.0117 (7)	0.0068 (7)

### Geometric parameters (Å, °)

**Table:**

C16—C17	1.522 (3)	C4—H4B	0.9600
C16—C15	1.525 (3)	C4—H4C	0.9600
C16—H16A	0.9700	C32—C31	1.528 (3)
C16—H16B	0.9700	C32—H32A	0.9700
C15—C14	1.525 (3)	C32—H32B	0.9700
C15—H15A	0.9700	C6—C3	1.544 (2)
C15—H15B	0.9700	C6—H6A	0.9600
C22—C21	1.535 (3)	C6—H6B	0.9600
C22—H22A	0.9600	C6—H6C	0.9600
C22—H22B	0.9600	C36—C31	1.527 (2)
C22—H22C	0.9600	C36—H36A	0.9700
C23—C21	1.535 (3)	C36—H36B	0.9700
C23—H23A	0.9600	C13—N1	1.461 (2)
C23—H23B	0.9600	C13—H13	0.9800
C23—H23C	0.9600	C31—N2	1.466 (2)
C9—O2	1.424 (2)	C31—H31	0.9800
C9—H9A	0.9600	C12—N1	1.280 (2)
C9—H9B	0.9600	C12—C11	1.460 (2)
C9—H9C	0.9600	C12—H12	0.9300
C24—C21	1.541 (3)	C2—C7	1.390 (2)
C24—H24A	0.9600	C2—C1	1.419 (2)
C24—H24B	0.9600	C2—C3	1.539 (2)
C24—H24C	0.9600	C20—C25	1.390 (2)
C34—C33	1.527 (3)	C20—C19	1.416 (2)
C34—C35	1.530 (3)	C28—C26	1.380 (2)
C34—H34A	0.9700	C28—C29	1.406 (2)
C34—H34B	0.9700	C28—H28	0.9300
C17—C18	1.530 (2)	C8—O2	1.373 (2)
C17—H17A	0.9700	C8—C10	1.378 (2)
C17—H17B	0.9700	C8—C7	1.404 (2)
C21—C20	1.536 (2)	C7—H7	0.9300
C35—C36	1.530 (2)	C19—O3	1.362 (2)
C35—H35A	0.9700	C19—C29	1.410 (2)
C35—H35B	0.9700	C25—C26	1.401 (2)
C33—C32	1.524 (3)	C25—H25	0.9300
C33—H33A	0.9700	C26—O4	1.370 (2)
C33—H33B	0.9700	C30—N2	1.278 (2)
C14—C13	1.529 (3)	C30—C29	1.461 (2)
C14—H14A	0.9700	C30—H30	0.9300
C14—H14B	0.9700	C10—C11	1.407 (2)
C18—C13	1.523 (2)	C10—H10	0.9300
C18—H18A	0.9700	C11—C1	1.411 (2)
C18—H18B	0.9700	C1—O1	1.3521 (19)
C5—C3	1.537 (2)	O3—H2	0.90 (3)
C5—H5A	0.9600	O1—H1	0.88 (3)
C5—H5B	0.9600	O4—C27	1.421 (2)
C5—H5C	0.9600	C27—H27A	0.9600
C4—C3	1.534 (2)	C27—H27B	0.9600
C4—H4A	0.9600	C27—H27C	0.9600
			
C17—C16—C15	111.12 (16)	C33—C32—C31	112.33 (14)
C17—C16—H16A	109.4	C33—C32—H32A	109.1
C15—C16—H16A	109.4	C31—C32—H32A	109.1
C17—C16—H16B	109.4	C33—C32—H32B	109.1
C15—C16—H16B	109.4	C31—C32—H32B	109.1
H16A—C16—H16B	108.0	H32A—C32—H32B	107.9
C16—C15—C14	111.66 (15)	C3—C6—H6A	109.5
C16—C15—H15A	109.3	C3—C6—H6B	109.5
C14—C15—H15A	109.3	H6A—C6—H6B	109.5
C16—C15—H15B	109.3	C3—C6—H6C	109.5
C14—C15—H15B	109.3	H6A—C6—H6C	109.5
H15A—C15—H15B	108.0	H6B—C6—H6C	109.5
C21—C22—H22A	109.5	C31—C36—C35	111.78 (14)
C21—C22—H22B	109.5	C31—C36—H36A	109.3
H22A—C22—H22B	109.5	C35—C36—H36A	109.3
C21—C22—H22C	109.5	C31—C36—H36B	109.3
H22A—C22—H22C	109.5	C35—C36—H36B	109.3
H22B—C22—H22C	109.5	H36A—C36—H36B	107.9
C21—C23—H23A	109.5	N1—C13—C18	110.41 (13)
C21—C23—H23B	109.5	N1—C13—C14	108.41 (14)
H23A—C23—H23B	109.5	C18—C13—C14	110.51 (15)
C21—C23—H23C	109.5	N1—C13—H13	109.2
H23A—C23—H23C	109.5	C18—C13—H13	109.2
H23B—C23—H23C	109.5	C14—C13—H13	109.2
O2—C9—H9A	109.5	N2—C31—C36	110.14 (14)
O2—C9—H9B	109.5	N2—C31—C32	108.00 (13)
H9A—C9—H9B	109.5	C36—C31—C32	110.85 (14)
O2—C9—H9C	109.5	N2—C31—H31	109.3
H9A—C9—H9C	109.5	C36—C31—H31	109.3
H9B—C9—H9C	109.5	C32—C31—H31	109.3
C21—C24—H24A	109.5	N1—C12—C11	122.93 (15)
C21—C24—H24B	109.5	N1—C12—H12	118.5
H24A—C24—H24B	109.5	C11—C12—H12	118.5
C21—C24—H24C	109.5	C7—C2—C1	117.59 (14)
H24A—C24—H24C	109.5	C7—C2—C3	121.64 (14)
H24B—C24—H24C	109.5	C1—C2—C3	120.76 (14)
C33—C34—C35	111.12 (15)	C25—C20—C19	116.99 (14)
C33—C34—H34A	109.4	C25—C20—C21	120.97 (15)
C35—C34—H34A	109.4	C19—C20—C21	122.04 (15)
C33—C34—H34B	109.4	C4—C3—C5	107.90 (14)
C35—C34—H34B	109.4	C4—C3—C2	111.20 (14)
H34A—C34—H34B	108.0	C5—C3—C2	110.35 (13)
C16—C17—C18	111.30 (15)	C4—C3—C6	107.98 (14)
C16—C17—H17A	109.4	C5—C3—C6	110.16 (15)
C18—C17—H17A	109.4	C2—C3—C6	109.21 (13)
C16—C17—H17B	109.4	C26—C28—C29	119.45 (14)
C18—C17—H17B	109.4	C26—C28—H28	120.3
H17A—C17—H17B	108.0	C29—C28—H28	120.3
C23—C21—C22	107.67 (16)	O2—C8—C10	125.03 (15)
C23—C21—C20	109.33 (14)	O2—C8—C7	115.45 (14)
C22—C21—C20	111.61 (15)	C10—C8—C7	119.53 (15)
C23—C21—C24	110.20 (15)	C2—C7—C8	122.81 (14)
C22—C21—C24	107.34 (15)	C2—C7—H7	118.6
C20—C21—C24	110.64 (14)	C8—C7—H7	118.6
C34—C35—C36	111.26 (15)	O3—C19—C29	119.94 (14)
C34—C35—H35A	109.4	O3—C19—C20	119.64 (14)
C36—C35—H35A	109.4	C29—C19—C20	120.42 (15)
C34—C35—H35B	109.4	C20—C25—C26	123.13 (15)
C36—C35—H35B	109.4	C20—C25—H25	118.4
H35A—C35—H35B	108.0	C26—C25—H25	118.4
C32—C33—C34	111.13 (15)	O4—C26—C28	125.54 (15)
C32—C33—H33A	109.4	O4—C26—C25	114.97 (15)
C34—C33—H33A	109.4	C28—C26—C25	119.50 (15)
C32—C33—H33B	109.4	N2—C30—C29	122.71 (15)
C34—C33—H33B	109.4	N2—C30—H30	118.6
H33A—C33—H33B	108.0	C29—C30—H30	118.6
C15—C14—C13	111.83 (15)	C28—C29—C19	120.51 (15)
C15—C14—H14A	109.3	C28—C29—C30	118.24 (14)
C13—C14—H14A	109.3	C19—C29—C30	121.19 (15)
C15—C14—H14B	109.3	C8—C10—C11	119.47 (14)
C13—C14—H14B	109.3	C8—C10—H10	120.3
H14A—C14—H14B	107.9	C11—C10—H10	120.3
C13—C18—C17	111.04 (14)	C10—C11—C1	120.86 (14)
C13—C18—H18A	109.4	C10—C11—C12	118.20 (14)
C17—C18—H18A	109.4	C1—C11—C12	120.91 (14)
C13—C18—H18B	109.4	O1—C1—C11	120.17 (14)
C17—C18—H18B	109.4	O1—C1—C2	120.15 (14)
H18A—C18—H18B	108.0	C11—C1—C2	119.68 (14)
C3—C5—H5A	109.5	C8—O2—C9	116.08 (14)
C3—C5—H5B	109.5	C19—O3—H2	102 (2)
H5A—C5—H5B	109.5	C1—O1—H1	103.8 (19)
C3—C5—H5C	109.5	C26—O4—C27	116.66 (14)
H5A—C5—H5C	109.5	C30—N2—C31	119.08 (14)
H5B—C5—H5C	109.5	C12—N1—C13	118.63 (14)
C3—C4—H4A	109.5	O4—C27—H27A	109.5
C3—C4—H4B	109.5	O4—C27—H27B	109.5
H4A—C4—H4B	109.5	H27A—C27—H27B	109.5
C3—C4—H4C	109.5	O4—C27—H27C	109.5
H4A—C4—H4C	109.5	H27A—C27—H27C	109.5
H4B—C4—H4C	109.5	H27B—C27—H27C	109.5
			
C17—C16—C15—C14	−54.2 (2)	C21—C20—C25—C26	−177.91 (15)
C15—C16—C17—C18	55.4 (2)	C29—C28—C26—O4	−179.58 (15)
C33—C34—C35—C36	−55.5 (2)	C29—C28—C26—C25	0.7 (2)
C35—C34—C33—C32	55.2 (2)	C20—C25—C26—O4	178.90 (15)
C16—C15—C14—C13	54.4 (2)	C20—C25—C26—C28	−1.3 (3)
C16—C17—C18—C13	−56.8 (2)	C26—C28—C29—C19	0.1 (2)
C34—C33—C32—C31	−55.0 (2)	C26—C28—C29—C30	−177.03 (14)
C34—C35—C36—C31	55.2 (2)	O3—C19—C29—C28	−179.94 (14)
C17—C18—C13—N1	176.14 (14)	C20—C19—C29—C28	−0.2 (2)
C17—C18—C13—C14	56.2 (2)	O3—C19—C29—C30	−2.9 (2)
C15—C14—C13—N1	−176.39 (15)	C20—C19—C29—C30	176.78 (14)
C15—C14—C13—C18	−55.3 (2)	N2—C30—C29—C28	179.60 (15)
C35—C36—C31—N2	−173.66 (14)	N2—C30—C29—C19	2.5 (2)
C35—C36—C31—C32	−54.19 (19)	O2—C8—C10—C11	178.57 (14)
C33—C32—C31—N2	175.06 (14)	C7—C8—C10—C11	−1.9 (2)
C33—C32—C31—C36	54.31 (19)	C8—C10—C11—C1	0.4 (2)
C23—C21—C20—C25	116.85 (18)	C8—C10—C11—C12	178.38 (14)
C22—C21—C20—C25	−2.2 (2)	N1—C12—C11—C10	177.62 (15)
C24—C21—C20—C25	−121.60 (17)	N1—C12—C11—C1	−4.4 (2)
C23—C21—C20—C19	−62.2 (2)	C10—C11—C1—O1	−178.49 (14)
C22—C21—C20—C19	178.83 (16)	C12—C11—C1—O1	3.6 (2)
C24—C21—C20—C19	59.4 (2)	C10—C11—C1—C2	2.1 (2)
C7—C2—C3—C4	0.0 (2)	C12—C11—C1—C2	−175.82 (14)
C1—C2—C3—C4	−178.69 (14)	C7—C2—C1—O1	177.60 (13)
C7—C2—C3—C5	−119.66 (16)	C3—C2—C1—O1	−3.6 (2)
C1—C2—C3—C5	61.61 (19)	C7—C2—C1—C11	−3.0 (2)
C7—C2—C3—C6	119.11 (16)	C3—C2—C1—C11	175.78 (13)
C1—C2—C3—C6	−59.63 (19)	C10—C8—O2—C9	−2.7 (2)
C1—C2—C7—C8	1.5 (2)	C7—C8—O2—C9	177.73 (15)
C3—C2—C7—C8	−177.24 (14)	C28—C26—O4—C27	−1.7 (2)
O2—C8—C7—C2	−179.48 (13)	C25—C26—O4—C27	178.01 (15)
C10—C8—C7—C2	0.9 (2)	C29—C30—N2—C31	−176.24 (14)
C25—C20—C19—O3	179.36 (14)	C36—C31—N2—C30	−118.58 (17)
C21—C20—C19—O3	−1.6 (2)	C32—C31—N2—C30	120.23 (16)
C25—C20—C19—C29	−0.4 (2)	C11—C12—N1—C13	176.05 (14)
C21—C20—C19—C29	178.70 (14)	C18—C13—N1—C12	127.20 (17)
C19—C20—C25—C26	1.2 (2)	C14—C13—N1—C12	−111.61 (17)

### Hydrogen-bond geometry (Å, °)

**Table:**

*D*—H···*A*	*D*—H	H···*A*	*D*···*A*	*D*—H···*A*
O1—H1···N1	0.88 (3)	1.77 (3)	2.5918 (19)	156 (3)
O3—H2···N2	0.90 (3)	1.73 (3)	2.5901 (19)	159 (3)
C5—H5B···O1	0.96	2.34	2.994 (2)	125
C6—H6B···O1	0.96	2.36	3.004 (2)	124
C23—H23B···O3	0.96	2.41	3.051 (2)	124
C24—H24B···O3	0.96	2.36	3.000 (2)	124

## References

[bb1] Bigi, F., Conforti, M. L., Maggi, R. & Sartori, G. (2000). *Tetrahedron*, **56**, 2709–2712.

[bb2] Darensbourg, D. J., Mackiewicz, R. M. & Billodeaux, D. R. (2005). *Organometallics*, **24**, 144–148.

[bb3] Farrugia, L. J. (1997). *J. Appl. Cryst.* **30**, 565.

[bb4] Farrugia, L. J. (1999). *J. Appl. Cryst.* **32**, 837–838.

[bb5] Gregson, C. K. A., Blackmore, I. J., Gibson, V. C., Long, N. J., Marshall, E. L. & White, A. J. P. (2006). *Dalton Trans.* pp. 3134–3140. 10.1039/b518266b 16786072

[bb6] Hiller, W., Nishinaga, A., Tsutsui, T. & Rieker, A. (1993). *Acta Cryst.* C**49**, 1357–1359.

[bb7] Hofsløkkn, N. U. & Skattebøl, L. (1999). *Acta Chem. Scand.* **53**, 258–262.

[bb8] Makio, H., Kashiwa, N. & Fujita, T. (2002). *Adv. Synth. Catal.* **344**, 477–493.

[bb9] Matsui, S. & Fujita, T. (2001). *Catal. Today*, **66**, 63–73.

[bb10] Matsui, S., Mitani, M., Saito, J., Tohi, Y., Makio, H., Matsukawa, N., Takagi, Y., Tsuru, K., Nitabaru, M., Nakano, T., Tanaka, H., Kashiwa, N. & Fujita, T. (2001). *J. Am. Chem. Soc.* **123**, 6847–6856.

[bb11] Matsui, S., Tohi, S., Mitani, M., Saito, J., Makio, H., Tanaka, H., Nitabaru, M., Nakano, T. & Fujita, T. (1999). *Chem. Lett.* pp. 1065–1066.

[bb12] Matsukawa, N., Matsui, S., Mitani, M., Saito, J., Tsuru, K., Kashiwa, N. & Fujita, T. (2001). *J. Mol. Catal. A*, **169**, 99-104.

[bb13] Parssinen, A., Luhtanen, T., Klinga, M., Pakkanen, T., Leskela, M. & Repo, T. (2005). *Eur. J. Inorg. Chem.* pp. 2100–2109.

[bb14] Saito, J., Mitani, M., Matsui, S., Tohi, Y., Makio, H., Nakano, T., Tanaka, H., Kashiwa, N. & Fujita, T. (2002). *Macromol. Chem. Phys.* **203**, 59–65.

[bb15] Sheldrick, G. M. (2008). *Acta Cryst.* A**64**, 112–122. 10.1107/S0108767307043930 18156677

[bb16] Stoe & Cie (2005). *X-AREA* Stoe & Cie, Darmstadt, Germany.

[bb17] Suzuki, Y., Tanaka, H., Oshiki, T., Takai, K. & Fujita, T. (2006). *Chem. Asia J.* **1**, 878–887. 10.1002/asia.200600256 17441131

[bb18] Tohi, Y., Nakano, T., Makio, H. Y., Matsui, S. K., Fujita, T. & Yamaguthi, T. (2004). *Macromol. Chem. Phys.* **205**, 1179–1186.

[bb19] Wang, R.-X., You, X.-Z., Meng, Q.-J., Mintz, E. A. & Bu, X.-R. (1994). *Synth. Commun.* **24**, 1757–1760.

